# Characterization, correction and *de novo* assembly of an Oxford Nanopore genomic dataset from *Agrobacterium tumefaciens*

**DOI:** 10.1038/srep28625

**Published:** 2016-06-28

**Authors:** Stéphane Deschamps, Joann Mudge, Connor Cameron, Thiruvarangan Ramaraj, Ajith Anand, Kevin Fengler, Kevin Hayes, Victor Llaca, Todd J. Jones, Gregory May

**Affiliations:** 1 DuPont Pioneer, Johnston, IA, USA; 2 National Center for Genome Resources, Santa Fe, NM, USA

## Abstract

The MinION is a portable single-molecule DNA sequencing instrument that was released by Oxford Nanopore Technologies in 2014, producing long sequencing reads by measuring changes in ionic flow when single-stranded DNA molecules translocate through the pores. While MinION long reads have an error rate substantially higher than the ones produced by short-read sequencing technologies, they can generate *de novo* assemblies of microbial genomes, after an initial correction step that includes alignment of Illumina sequencing data or detection of overlaps between Oxford Nanopore reads to improve accuracy. In this study, MinION reads were generated from the multi-chromosome genome of *Agrobacterium tumefaciens* strain LBA4404. Errors in the consensus two-directional (sense and antisense) “2D” sequences were first characterized by way of comparison with an internal reference assembly. Both Illumina-based correction and self-correction were performed and the resulting corrected reads assembled into high-quality hybrid and non-hybrid assemblies. Corrected read datasets and assemblies were subsequently compared. The results shown here indicate that both hybrid and non-hybrid methods can be used to assemble Oxford Nanopore reads into informative multi-chromosome assemblies, each with slightly different outcomes in terms of contiguity and accuracy.

Second-generation DNA sequencing technologies have revolutionized the field of genomics^1^,^2^ and given researchers access to unprecedented amounts of sequencing data. Most of those platforms use a massively parallel sequencing-by-synthesis approach, where millions of clustered amplicons serve as templates for reactions that produce reads up to a few hundred bases in length^3^,^4^. While short read lengths are not considered an impediment to the study of small genomic structural variations, such as SNPs, epigenomic variation, gene expression profiling or genotypic determination^5^,^6^,^7^,^8^, they generally make it challenging to solve complex genome assembly problems such as long repetitive sequences or segmental duplications. As a result, they do not generally provide a complete assembly of a particular genome. A third generation of sequencers, namely single-molecule long-read DNA sequencers has proven to be a useful alternative to short read DNA sequencers. The Single Molecule Real Time (SMRT) sequencing platform from Pacific Biosciences (“PacBio”) utilizes a sequencing-by-synthesis approach, where labeled nucleotides are incorporated into native DNA strands in a real-time fashion^9^. As sequencing occurs on single molecules instead of clustered amplicons, sequencing length limitations inherent to second-generation sequencers are virtually eliminated. As a result, the SMRT platform is capable of producing individual sequencing reads up to ~40 Kb in length, thus allowing complex repetitive regions to be contained within a single sequencing read. In addition, hairpin adapters are ligated at both ends of double-stranded DNA, thus creating a circular template that can be sequenced through multiple passes of the DNA polymerase to build a more accurate consensus read^10^,^11^. Assemblies can be performed with SMRT data alone, or in combination with short-read sequencing data. The choice of a “hybrid” or “non-hybrid” assembly strategy depends of several factors, including the size and complexity of the genome being sequenced, but also the costs and analysis to be expected from the project. Nevertheless, the use of long reads on microbial and eukaryotic genomes has demonstrated the possibility of generating very large contiguous sequences, or “contigs”, often encompassing large repeated and complex elements^12^,^13^,^14^. In many microbial genome assemblies, the length of those contigs has often reached the length of chromosomes or plasmids present in the microbial cell^15^.

In early 2014, Oxford Nanopore Technologies released another single-molecule real-time sequencing device, called the MinION. The MinION is a portable nanopore-based sequencer connected to a desktop through a USB 3.0 interface. Sequencing involves measuring changes in ionic current when DNA strands translocate through protein nanopores in an insulated membrane. During translocation, bases within a DNA strand block the flow of ions to a different degree, altering the current and creating signature signals that are identified electronically and translated into specific base calls. Because incorporation of nucleotides is not involved in the sequencing process, read length, in theory, is only limited by the length of the DNA strand translocating through the pore, and sequences >100 Kb in length have already been reported. A hairpin adapter ligated to the opposite end of the double-stranded DNA template allows the sense and antisense strands to be sequenced consecutively. The resulting data are aligned to create a higher quality consensus “2D” sequence. While early reports^16^ indicated a high error rate for MinION reads (~35%), their accuracy has improved over time as Oxford Nanopore has released new versions of its base calling software and nanopore chemistry. Recent reports have suggested the MinION can be used for generating *de novo* assemblies of several organisms. However, due to the relatively high error rates exhibited by the technology, those assembly projects required using the nanopore reads for scaffolding only^17^,^18^, or required an initial error correction step, where MinION reads were aligned to themselves or to Illumina reads to correct miscalls prior to assembly^19^. Loman *et al*.^20^ successfully demonstrated that Oxford Nanopore data alone (~30X genome coverage) could be used, after read correction using multiple alignments between overlapping reads to generate a consensus sequence, to create a single-contig *de novo* assembly of the *E. coli* genome, with a percentage identity of 98.4% compared to the finished reference assembly. This assembly was performed using a series of software tools specifically developed for extracting FASTA sequences from raw signals (“poretools”), correcting overlapping reads (“nanocorrect”) and polishing the assembly (“nanopolish”). Similarly, Goodwin *et al*.^19^ assembled the *S. cerevisiae* genome, using a software tool specifically designed for hybrid error correction with Illumina data, and reported an assembly of the corrected Oxford Nanopore reads (corresponding to ~20X coverage of the genome), into 108 contigs, with an N_50_ of 678 Kb and a percentage identity of 99.88% to the reference assembly.

In the present study, the sequencing accuracy of individual MinION 2D reads generated from the strain LBA4404 of the plant pathogen *Agrobacterium tumefaciens* was evaluated, following the alignment of the 2D reads to an internal Illumina and PacBio-based reference assembly. The sequence accuracy of the *de novo* assemblies performed with corrected 2D reads also was determined by way of comparison with the internal reference. *De novo* assemblies were performed with the hierarchical assembly pipeline PBcR^21^ and the recently released canu *de novo* assembler^22^, which specializes in assembling single-molecule sequencing data with relatively high error rates, such as PacBio or Oxford Nanopore data. PBcR has proven to be an excellent tool for assembling PacBio SMRT data for small and large genomes^23^, using either a hybrid or non-hybrid assembly approach. A goal of this study was to demonstrate the efficacy of the pipelines on microbial MinION read correction and assembly, in relation to previously published tools such as nanocorr^19^, nanocorrect^20^ or SPAdes^24^. *Agrobacterium tumefaciens* strain LBA4404 was chosen as a model system for this study because it is a preferred strain used in *Agrobacterium*-mediated plant transformation^25^. Its genome is almost identical to the genome of its immediate precursor LBA4213^26^, and an Illumina-based draft genome assembly, currently made up of 39 contigs, is available for further comparison (JMKN01000001 to JMKN01000039). Its genome consists of a circular chromosome, a linear chromosome, a megaplasmid (“At plasmid”) and a tumor-inducing (Ti) plasmid, and is therefore more complex than most microbial genomes made of a single circular chromosome. The results shown here provide more information on the Oxford Nanopore sequencing technology and confirm that PBcR and canu can effectively correct MinION 2D reads and assemble them into a multi-chromosomal microbial genome, with a small number of contigs and very high sequence accuracy.

## Results

### MinION sequencing and base calling

A total of six LBA4404 whole-genome shotgun libraries were sequenced on the MinION using the R7.3 chemistry. Genomic DNA was randomly sheared, targeting a mean fragment size of ~8 Kb prior to library construction and sequenced on a “classic” MinION. One flow cell was used for each library, for a total of six flow cells. Sequencing from all six runs produced a total of 32,287 “passed” 2D reads (using internal quality criteria determined by the cloud-based Metrichor™ agent), out of which 1,875, corresponding to a control DNA sample added during library construction, were removed. The remaining 30,412 2D reads were included in all subsequent analyses, had a total yield of 171,499,518 bps, corresponding to ~31X coverage of the genome, with the longest read at 32,420 bps and a N_50_ read length of 6,500 bps. Lower quality 1D reads and “failed” 2D reads (where sense and antisense strand sequences can’t be aligned to generate a consensus 2D base call) generated during the sequencing runs were not used in subsequent analyses.

**Figure 1:**
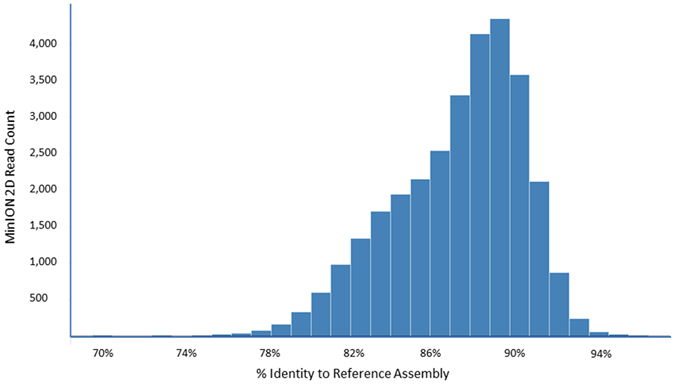
MinION sequence accuracy histogram. X-axis: percentage identity of the portion of the 2D reads aligning to the reference assembly. Y-axis: raw counts of MinION 2D reads aligning to the reference assembly and clustered with a given identity percentage point. Percentages were determined after alignment of raw 2D reads to the reference assembly using BWA –MEM and automated retrieval of percentage identity from the BWA –MEM output.

In order to assess the mapability of MinION 2D reads, the four contigs from an Illumina and PacBio-based reference assembly of the *A. tumefaciens* LBA4404 genome were used as references against which MinION 2D reads were aligned. The reference assembly was generated with Illumina paired-end and mate-pair data, complemented with PacBio data and further curated with public Illumina assembly data. The four contigs (Table 1) consisted of a circular chromosome (2,772,940 bps), a linear chromosome (2,098,034 bps), a large “At” plasmid (556,650 bps) and a small “Ti” plasmid (109,974 bps). Alignments were performed using BWA –MEM^27^ and results indicate that all 30,412 2D reads aligned to the reference. 94.7% of the alignments covered at least 95% of the corresponding MinION reads, with the lowest alignment covering 39.9% of the read. Sequence identity of the MinION 2D reads aligned to the reference assembly ranged from 69.7% to 96.3%, averaging 87.2% (Fig. 1).

**Figure 2:**
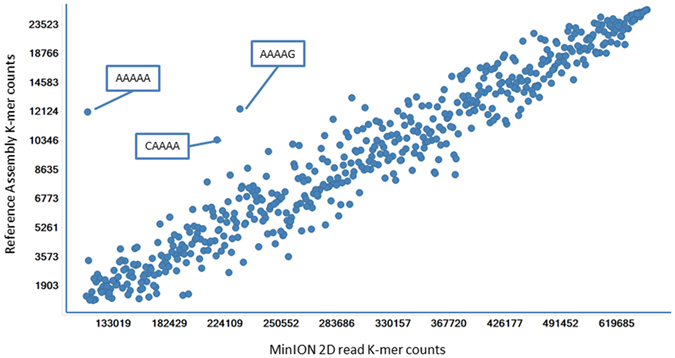
MinION K-mer retrieval. X-axis: counts of each individual 5-mers in the MinION 2D read dataset; Y-axis: counts of each individual 5-mers in the reference assembly.

Base calling with the MinION base calling software was performed by reading signals originating from 5 adjacent bases. Therefore, pentanucleotide sequences (“5-mers”) were extracted *in silico* from the MinION 2D read datasets to assess the possibility of a base calling bias. Their absolute counts were compared to the counts of similar 5-mers in the reference assembly. As shown in Fig. 2, “AAAAA” was the most under-represented 5-mer in the MinION dataset relative to its actual count in the reference assembly. A similar pattern was observed for other AT-rich 5-mers, including “CAAAA”, and “AAAAG”. Those results can be explained by the fact that the MinION relies on changes in electric signals to detect transitions between bases. Homopolymers translocate through the pore with no changes in such signal, and the number of nucleotides in the homopolymer must be inferred from the detectable signal, introducing random indels that can be eliminated only by increasing sequencing coverage^28^.

Sequencing coverage for each component of the genome was as follows: 6.1X for the Ti plasmid; 22X for the At plasmid; 29.9X for the circular chromosome and 30.8X for the linear chromosome. The lower overall sequencing coverage of the Ti plasmid could be related to the method used to prepare DNA prior to sequencing. However, to assess the possibility of a process bias specific to the Ti plasmid, analysis of the GC-content of all four components of the genome was performed by determining 5-mer counts in the reference assembly. The results indicated that, while all possible 5-mer combinations are present in the Ti plasmid, the ones with both high and low GC-contents were more prominent in the Ti plasmid than in the other elements of the genome, suggesting a possible bias during library construction (i.e., shearing) or sequencing of the plasmid. A total of 6,807 nucleotides from the reference assembly did not have any sequencing coverage, while 117,654 nucleotides exhibit <10X sequencing coverage (Table 1). A vast majority of those 117,654 bps originated from the small Ti plasmid. The analysis of 5-mers surrounding the missing 6,807 nucleotides from the MinION 2D read dataset (where the missing nucleotide was placed in the middle of the 5-mer) indicated that one-base deletions impacted equally all four nucleotides, and was contained within 994 distinct 5-mer sequences, out of a theoretical maximum of 1,024.

**Figure 3:**
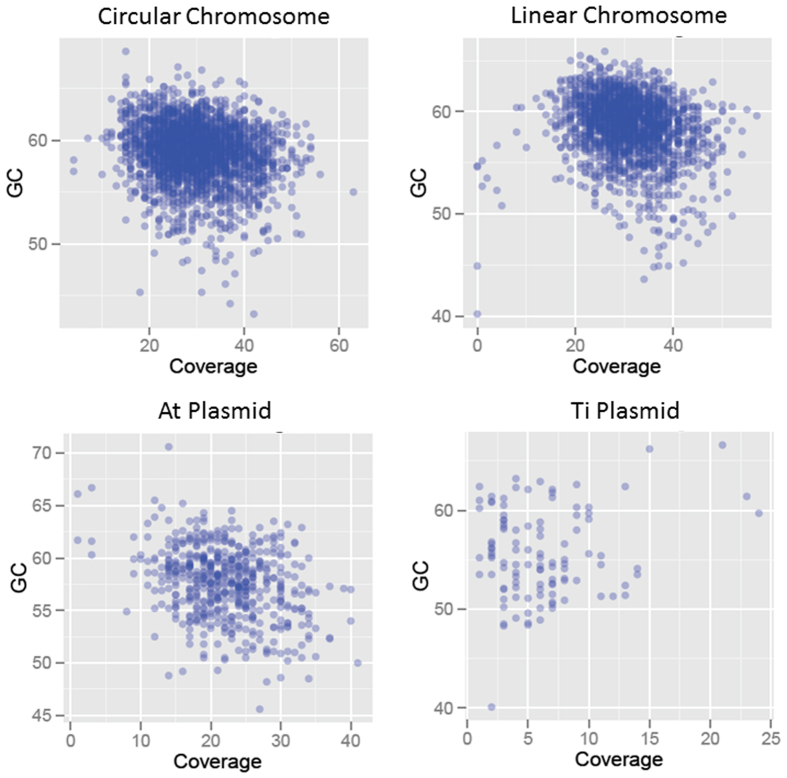
MinION sequence distribution in relation to G+C content. Sequencing coverages of the reference assembly by individual 2D reads were plotted for all four components of the *Agrobacterium tumefaciens* strain LBA4404 genome against the G+C content of the same individual’s 2D reads. G+C content for 2D reads were determined incrementally using a 1-Kb window size.

Finally, the average G+C content of the *Agrobacterium* reference assembly was 58.5% while the average G+C content of all individual 2D MinION reads was 55.8%. To assess a possible correlation between the G+C content of the genome and the ability of the MinION to sequence regions with high G+C content, sequencing coverage was graphed in relation to the G+C content of the reference assembly for all four elements of the *Agrobacterium* genome (Fig. 3). The data showed a relatively weak correlation between high G+C content and sequencing coverage, with high sequencing coverage in three of the four elements (>40X in linear and circular chromosomes, >30X in megaplasmid) mainly occurring in regions of the reference assembly with a G+C content <60% (the relatively low sequencing coverage of the Ti plasmid didn’t allow for any conclusive statement about G+C content and its relation to coverage for that particular plasmid). While the relatively uniform sequencing coverage and G+ C distribution across the reference assembly doesn’t rule out the possibility of a G+C bias inherent to the technology, or of a bias more pronounced in 1D and failed 2D reads (that were not part of this analysis), the slightly lower average G+C content of individual 2D MinION reads could also originate from higher rates of base calling errors in G+C-rich MinION reads.

To investigate this possibility, substitutions were called in individual MinION 2D reads aligned to the reference assembly, using the variant calling model in SAMtools/bcftools^29^,^30^,^31^,^32^,^33^ with default quality filtering (Q >= 13) and the additional requirement that a nucleotide position kept after quality filtering exhibits at least 6X MinION 2D read coverage, well below the average MinION 2D sequencing coverage for the entire genome. A total of 150 “minor” substitutions (with a frequency less than half of all base calls at that position) were found post-quality filtering using all 30,412 2D reads. They were distributed equally between the two chromosomes (70 on the linear chromosome, 73 on the circular chromosome) and 7 additional substitutions were found on the At plasmid (the absence of substitutions on the Ti plasmid can be explained by its low overall sequencing coverage). A vast majority of the 150 calls involved C’s (72/150) or G’s (65/150) in the reference assembly sequence: G-to-A (48/150) and C-to-T (54/150) transitions were the most prevalent, followed by G-to-T (14/150) and C-to-A (14/150) transversions. Altogether, 130 G/C-to-A/T substitutions and only 11 A/T-to G/C substitutions were detected. This ~11.8-fold difference is much higher than the difference expected from the G+C content distribution of the reference assembly (which stands at ~1.4-fold difference). Taken together, these results suggest a higher likelihood of base calling errors where G’s or C’s in the reference assembly are substituted to A’s and T’s in the MinION 2D reads. To confirm, SAMtools/bcftools was re-run without the >6X coverage filter, and a similar trend was observed, where 1,728 out of a total of 1,809 detected substitutions involved C’s or G’s in the reference assembly.

Using the same 6X MinION read coverage requirement after quality filtering, a total of 2,090 indels were detected. Deletions in the MinION read sequences ranged from 1 base to 36 nucleotides in length, while the largest insertion was 10 nucleotides in length. Interestingly, several sequencing variations caused by indels matching the same sequence motif in the reference assembly were conserved across the genome. As an example, “CGAGA” in the reference assembly was translated into “CGA” (with a 2 bp “GA” deletion) in MinION 2D reads mapping to 8 separate regions of the two chromosomes and the At plasmid.

### MinION 2D read correction

Due to their relatively high error rates, error correction of ONT reads has been shown to be a critical component for generating higher quality consensus sequences and facilitating *de novo* assembly. Corrections can occur by comparing with other reads generated from high-density sequencing platforms, including the Illumina short read platform^19^. Loman *et al*.^20^ also have shown that corrections based on detecting overlaps between MinION reads alone were sufficient to provide high quality corrected reads prior to assembly of a bacterial genome. Five correction methods were tested in this study: (1) Illumina-based correction with PBcR; (2) Illumina-based correction with nanocorr^19^; (3) self-correction with PBcR; (4) self-correction with canu; and (5) self-correction with PoreSeq^34^. Self-correction with nanocorrect was initiated but not pursued because of processing issues. All 30,412 MinION 2D reads were used for correction. For Illumina-based correction with PBcR, the original PBcR parameters, designed for PacBio reads were used. It was speculated that the use of those parameters on MinION “passed” 2D reads would not impact the assembly in a negative manner, as those reads exhibited average error rates that closely matched PacBio thresholds for correction. Illumina-based corrections were performed by aligning 8.9 million 150 bp Illumina paired reads (corresponding to >250X coverage) generated from the same whole genome *Agrobacterium* LBA4404 DNA sample. Corrections were made in accordance with the Illumina base call mapping to the same region of DNA as the erroneous MinION base call. Subsequent trimming and splitting of the corrected reads were performed whenever a gap occurred in the Illumina read tiling path.

All five correction processes are summarized on Table 2. Results show that Ilumina-based correction with PBcR and self-correction with canu provided the best results, in terms of sequence identity to the reference assembly and percentage of reads with at least 99% identity between the reference assembly and the aligned portion of the read. Alignments to the reference were performed using BWA –MEM, which computed all partial alignments to the reference. 99.1% of the alignments after correction with PBcR contained at least 95% of the Illumina-corrected reads (including 93.4% of the alignments covering 100% of the reads). Similar analysis with the canu corrected set indicated that 99.7% of the alignments contained at least 95% of the self-corrected reads (including 82.3% of the alignments covering 100% of the reads).

The use of PoreSeq on the *Agrobacterium* MinION 2D read dataset was tested. PoreSeq is an algorithm that uses a statistical model based on the probability of observing an ionic level for a given 5-mer sequence and using the current ionic data for all reads of the same region of DNA to determine the maximum-likelihood sequence for that region. Unfortunately, the analysis of the 30,412 MinION 2D reads led to the generation of only 707 corrected reads after being run for nearly 2 months (further inquiries into the process indicated that it was not possible to run it on multiple threads or to subset and distribute the data, as it required to be pointed to a folder with the entire read dataset. It must be noted however that the underlying algorithm is valid and a new implementation of PoreSeq was released soon after data were gathered for this study).

**Figure 4:**
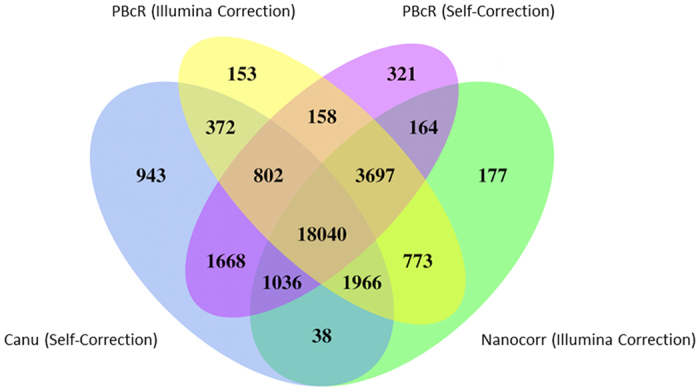
Venn diagram comparing MinION 2D read correction processes. Four of the five corrected datasets were compared, including Illumina-corrected reads generated with PBcR (yellow) and nanocorr (green), and self-corrected reads generated with PBcR (purple) and canu (blue). Numbers show the number of 2D corrected reads located at the intersection of 2 or more datasets, or unique to a particular dataset.

To compare different read correction methods, a Venn diagram was generated comparing all corrected datasets (Fig. 4). All corrected reads were traced back to the original MinION 2D reads they originated from, and overlaps between methods were computed accordingly. Results showed that a majority of all reads (18,040 reads) were shared between the four datasets (incorporation of the 707 PoreSeq corrected reads indicated that a total of 399 reads were conserved among all five datasets). Each method had a significant number of reads that were shared either with some other datasets, or were unique to the method being used to generate them. Such results could be explained by some corrections actually introducing errors in the reads. To evaluate the possibility, reads from all five corrected sets that had not been split upon correction were compared before and after correction for their identity to the reference assembly. Results indicated that none of these corrected reads exhibited a lower sequence identity to the reference after correction. Though it is possible that correction has introduced some errors at isolated nucleotides, it clearly improved the accuracy of the reads overall.

### *De novo* Assemblies

Five separate assemblies were performed: two non-hybrid assemblies with the self-corrected read sets generated with canu and PBcR, and three hybrid assemblies with the Illumina-corrected read sets generated with nanocorr and PBcR. Since canu is designed to work directly with high-noise single-molecule sequencing reads, the Illumina-corrected read set generated with PBcR was used for both the PBcR and canu hybrid assemblies. The entire self-corrected read set was used to generate the non-hybrid assemblies while only the top 40% of the Illumina-corrected reads, based on lengths, were used for the hybrid assemblies (those reads varied in size between 1,323 and 26,564 bps, and yielded approximately 132 Mbps of data). The PBcR hybrid and non-hybrid assemblies were performed with the default Celera Assembler contained within the PBcR pipeline. Scaffolding and polishing of all assemblies was performed with SSPACE^35^ and Pilon^36^. A statistical summary of all assemblies is presented in Table 3. Results indicate that, while the two non-hybrid assemblies have a lower number of contigs overall, the three hybrid assemblies exhibit slightly higher sequence accuracy after BLASTN alignment to the reference assembly.

**Figure 5:**
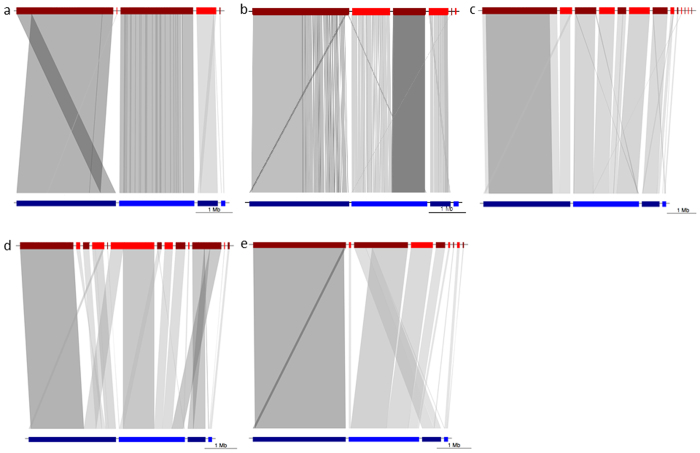
BLASTN comparisons of MinION assemblies to the reference assembly. (Top) Contigs from various MinION 2D read assemblies are shown in red; (Bottom) Contigs from the reference assembly are shown in blue (listed in the following order, from left to right: circular chromosome, linear chromosome, At plasmid, Ti plasmid). Ribbon plots are shown where assemblies were aligned and compared to the reference assembly using BLASTN. (**a**) canu non-hybrid assembly; (**b**) PBcR non-hybrid assembly; (**c**) PBcR hybrid assembly; (**d**) canu hybrid assembly; (**e**) SPAdes hybrid assembly. Scale is shown in Mbps. The discontinued homology to the circular chromosome shown in (**a**) likely is due to a “wraparound” effect due to the circular nature of the chromosome.

The canu (Fig. 5a) and PBcR (Fig. 5b) non-hybrid assemblies included 5 and 6 contigs respectively (Table 3), with a total contig length after polishing corresponding to the equivalent of 98.5% (canu) and 97.5% (PBcR) of the reference genome. Interestingly, the small Ti plasmid (109,974 bps) was virtually absent from the non-hybrid assemblies (only one small contig mapping to the plasmid was detected after alignment). The lack of contig coverage for the Ti plasmid could be explained by the low initial MinION 2D read coverage (6.1X), and the need, potentially, for a minimum read coverage threshold for effective self-correction and/or *de novo* assembly. In addition, relaxing gap penalty parameters in BLASTN led to a more contiguous alignment of the contigs to the reference assembly, suggesting that at least some of the observed discrepancies were small gap variants.

The PBcR hybrid assembly (Fig. 5c) was made of 22 contigs (Table 3), totaling 5,499,815 bps before polishing. In spite of a larger number of contigs than in the non-hybrid assemblies, 5,178,887 bps, or >94% of the genome, was contained within the 10 largest contigs. BLASTN alignment to the reference assembly indicated that the hybrid assembly (after read correction) contained a total of 60 single-base substitutions and 17 small gaps. The analysis of the gap regions in the reference assembly did not indicate any unusual differences in G+C content when compared to regions adjacent to the gaps. Scaffolding and polishing of the assembly with SSPACE and Pilon decreased the number of contigs to 13 (Table 3) and further increased sequence identity to the reference to >99.87%. One of the resulting contigs after polishing aligned to the At plasmid and the Ti plasmid (Fig. 5c). Further analysis indicated that this result was in fact due to an alignment artifact caused by a nearly identical 11-Kb region shared by the two plasmids.

Polishing of the canu hybrid assembly (Fig. 5d) decreased the number of contigs from 15 to 13, but did not increase sequence identity to the reference (Table 3). BLASTN alignment to the reference showed that one of the largest contigs aligns to two separate regions of the genome, located, respectively, on the circular and linear chromosomes. The connection on this particular contig occurred at a position covered by a single Illumina-corrected read. Interestingly, the related uncorrected MinION 2D read aligned only to the circular chromosome whereas the three Illumina-corrected subreads derived from it aligned to both chromosomes, with one of the corrected subreads creating this artificial connection. An additional chimera was found in this assembly, connecting the linear chromosome with the At plasmid. This connection was supported by one individual corrected subread on the linear chromosome exhibiting a very small overlap with four subreads at the end of a contig mapping to the At plasmid.

Finally, the SPAdes hybrid assembly (Fig. 5e) included the use of nanocorr for Illumina-based correction, followed by *de novo* assembly with the SPAdes software^24^, and led to the highest sequence identity percentage to the reference (>99.97%). One contig however appeared to be chimeric, with two adjacent regions aligning to the linear chromosome and the At plasmid, respectively. Further analysis indicated the presence of a ~200 bps region covered by 13 corrected subreads and connecting two distinct contigs. The presence of multiple sequencing variants in those 13 subreads in the region overlapping with one of the two contigs suggests the presence of repetitive sequences that may have led to the artefactual connection.

## Discussion

The present study demonstrates that corrected MinION 2D reads can be assembled into a multi-chromosome bacterial genome using hybrid and non-hybrid assembly software designed specifically for high-noise single molecule sequencing reads. An internal reference genome assembly generated for the *Agrobacterium* strain LBA4404 was used for direct comparison and assessment of the whole genome MinION 2D reads and their assemblies.

The existence of a G+C bias in this particular dataset was mostly related to the existence of a base calling error bias, favoring G/C-to-A/T substitutions, thus lowering artificially the overall 2D read G+C content. Most errors in this particular dataset were multi-allelic variants that were corrected through alignments of Illumina reads or other MinION reads. The existence of more systematic errors in the 1D read and failed 2D read datasets that were produced as part of this study should not be ruled out but the present data suggest that focusing only on the passed 2D data for *de novo* assembly could facilitate read correction and, subsequently, *de novo* assembly.

Both hybrid and non-hybrid read correction both lead to highly accurate corrected reads. The PBcR Illumina-based correction and canu self-correction processes provided corrected reads with the highest accuracies. However, it must be noted that Illumina-based correction of this particular dataset led to the creation of smaller split reads and their subsequent insertion into potentially chimeric contigs. On the other hand, some of the missing sequences in the non-hybrid assemblies mapped to the small Ti plasmid and it can be speculated that the MinION 2D reads mapping to this plasmid had an initial sequencing coverage that was too low for an efficient self-correction process. The use of either a hybrid or non-hybrid process for read correction should be dictated mostly by the size and complexity of the template being sequenced: Illumina-based correction of MinION reads generates more accurate reads, but it might not always be a realistic option (assuming, for example, a large and complex genome, where short Illumina reads may not necessarily align uniquely) and the correction process itself could create reads mapping to multiple regions of the genome.

Illumina-based correction of individual 2D reads led to assemblies with slightly higher sequence accuracy (based on their sequence identity to the reference) than self-correction. According to Sović *et al*.^37^, non-hybrid assembly methods require higher sequencing coverage than hybrid method for efficient *de novo* assembly of a bacterial genome. It remains to be determined whether the slightly lower accuracy of the self-corrected assemblies could be offset in future studies with higher sequencing coverage. Also, the total contig sizes shown in Table 3 suggest that polishing had a bigger impact on sequence identity and genome sequencing coverage for the non-hybrid assemblies but had less of an effect for the hybrid assemblies.

The fact that the *Agrobacterium* assemblies presented here did not reach completion (i.e., one contig per chromosome or plasmid) could be related to several factors, including the size and complexity of the *Agrobacterium* genome, the overall MinION 2D sequencing coverage and the number of corrected 2D reads used in the assembly process, or the choice of tools used for correction and *de novo* assembly. As Illumina-based correction of Oxford Nanopore reads prior to assembling large and complex genomes is expected to be quite inefficient, one logical progression in assessing the Oxford Nanopore technology will be to determine whether large genome assemblies, or at the very least the assembly of their non-repetitive fractions, can be performed using Oxford Nanopore data alone, corrected through self-correction or through the use of complementary algorithms and software, designed for working with error models specific to nanopore sequencing chemistries. This study can be viewed as a first step toward demonstrating the ability of single-molecule assemblers to process Oxford Nanopore read data into an informative multi-chromosome genome assembly.

## Materials and Methods

### Sample preparation

Genomic DNA from *Agrobacterium tumefaciens* strain LBA4404 was extracted using the Gentra Puregene Yeast/Bacterial kit (Qiagen) and quantified using the PicoGreen dsDNA Assay kit (Thermo Fisher).

### MinION library preparation

One microgram of purified genomic DNA was randomly sheared to an average of ~8 Kbps using a Covaris g-TUBE (Covaris). MinION library preparation was performed using components from the Genomic DNA Sequencing Kit (Oxford Nanopore Technologies) as follows. After shearing, DNA was subjected to a PreCR treatment (NEB) to repair potential nicks, cleaned up using 1x AMPure XP beads (Beckman Coulter) and eluted in 80 μl of nuclease-free water. End-repair was then performed after adding 5 μl control “CS” DNA (Oxford Nanopore Technologies), using the NEBNext End Repair Module (NEB). After purification with 1x volume AMPure XP beads (Beckman Coulter) and elution in 25 μl of nuclease-free water, dA-tailing was performed with the NEBNext dA-Tailing Module (NEB). For the adapter ligation step, the dA-tailed DNA was mixed with 10 μl Adapter Mix (Oxford Nanopore Technologies), 2 μl HP Adapter (Oxford Nanopore Technologies) and 50 μl Blunt/TA Ligase Master Mix (NEB) and incubated for 10 minutes at room temperature. Prior to ligation clean-up, 10 μl His-tag dynabeads (Life Technologies) were washed in 1x wash buffer (Oxford Nanopore Technologies) and resuspended in 100 μl 2x wash buffer, to which the ligation mix (100 μl) was added and incubated for 5 minutes. After washing the beads twice with 1x wash buffer, the DNA was eluted in 25 μl elution buffer (Oxford Nanopore Technologies) and quantified using a Qubit (Life Technologies) to estimate the total amount of DNA prior to loading the MinION.

### MinION sequencing and base calling

For loading, 6 μl of the final library was diluted in 140 μl EP Buffer (Oxford Nanopore Technologies) and 4 μl Fuel Mix (Oxford Nanopore Technologies) and loaded onto a MinION R7.3 Flow Cell. Sequencing was performed for 48 hours with reloading of the flow cell with the same loading mix after 24 hours.

Base calling of the raw MinION data was performed with the cloud-based Metrichor™ Agent. Base calling data were retrieved in the fast5 format and automatically sorted into two folders, one containing full-length “passed” 2D reads (based on alignment of two 1D reads and base calling of the resulting consensus) and the other containing partial “failed” 2D reads, template 1D reads and complement 1D reads.

### Read assessment

To assess potential bias in pentanucleotide base-calling, 5mers (both strands) were extracted using Jellyfish (v. 1.1.10)^38^. Frequencies of each 5mer were compared between raw reads and the reference and plotted in a scatterplot. The length and GC content of reads was obtained using the infoseq program in the EMBOSS package (6.4.0)^39^. The GC content of assemblies was obtained using a custom perlscript to obtain a GC datapoint every 1 kb (window size = 1 kb).

### Alignments

Alignment of reads, both raw and corrected, to the reference was done using BWA-MEM (v. 0.7.12) with parameters adjusted for the Oxford Nanopore 2D read type (“-x ont2d”) which implements several parameter changes (“-k14 -W20 -r10 -A1 -B1 -O1 -E1 -L0”). The percent read aligned and the percent identity of all primary alignments, which are only soft-clipped in BWA-MEM, were calculated as follows. The percent read aligned was calculated as the aligned read length divided by the total read length where the aligned read length was calculated as the length of the query minus soft-clipped bases. The percent identity was calculated as the number of matches (obtained by adding up all numbers in the MD tag) divided by the aligned read length.

### Coverage and variant calling

To find regions of low or no coverage and to compare coverage to GC levels, the coverage of each nt was generated from the alignment files (see above) using the Bio::DB::Sam perl module and a custom perlscript. For comparisons to GC content, coverage datapoints every 1 kb were used to match the 1 kb GC windows that were generated (see above).

Variants were called based on the alignments described above. Variant calling used SAMtools v. 1.1 for reference indexing (faidx) and read pileup (mpileup) and bcftools v. 1.2 for variant calling (call), filtering (filter) and querying (query). The mpileup default of skipping bases with a quality score less than 13 was used. This was followed by removal of any nucleotide position that had less than 6X quality-filtered coverage unless otherwise noted. Variants were called with the multi-allelic calling model.

### Read correction and assemblies

PBcR (the PacBio Corrected Reads Pipeline) from the 8.3rc2 version of the wgs-assembler package was run using the original PacBio parameters. This assembler is specifically made for high noise, long read data. The minimum length of sequences to correct was set to 500 bps and the number of partitions for consensus to 200. For Illumina read correction, fastqToCA was first run to create a frg file from the Illumina paired end reads and the original PacBio parameters were used (decrease merSize: merSize = 14). For all other assemblies, Oxford-recommended parameters were used. The merSize was again decreased to 14. The Falcon sense consensus was used but adjusted for lower quality reads (falconForce = 1,falconOptions = –max_n_read 200–min_idt 0.50–output_multi–local_match_count_threshold 0). The assembly paramaters were adjusted to overlap at a higher error rate than for PacBio (asmOvlErrorRate = 0.3, asmUtgErrorRate = 0.3, asmCgwErrorRate = 0.3, asmCnsErrorRate = 0.3, asmOBT = 0, batOptions = -RS –CS, utgGraphErrorRate = 0.3, utgMergeErrorRate = 0.3).

Canu version 1.0 was run using default parameters in nanopore-raw mode on 2D Oxford Nanopore reads to create a *de novo* assembly and correction of the raw 2D read input. Canu was also employed to perform assembly of 2D reads that had been corrected with PBcR using the Illumina data. For this, canu was run in nanopore-corrected mode with default parameters.

Nanocorr read correction was distributed in an SGE environment using default parameters following the README at https://github.com/jgurtowski/nanocorr. No reference was given and hence no alignment of corrected reads to the reference was done. SPAdes version 3.5.0-Linux used both the Nanopore and the Illumina reads. Default parameters were used.

PoreSeq read correction runs within a virtual environment using only nanopore fast5 files. It models uncertainty sources from nanopore sequencing using data from multiple reads derived from the same region to correct reads. It was run in default mode. No parameterization was possible.

### Plotting and visualization

Plotting was done in R using ggplot2^40^ and genoplotr^41^. Visualization of alignments and variants was done using the Integrative Genomics Viewer (Broad Institute).

### Assembly scaffolding and polishing

Scaffolding was performed utilizing the SSPACE long read scaffolder and the corrected read set from PBcR (http://www.baseclear.com/genomics/bioinformatics/basetools/SSPACE-longread). SSPACE long employs the BLASR aligner, which is used to align the corrected long read set to the PBcR assembly. Several parameterizations of varying stringencies on length of alignment, joins required for scaffolding, link-ratio, identity of alignment, and gap length were considered for this program. The final set of parameters utilized was -a 1500 -g -7500 -l 3, meaning a minimum alignment of 1500 bases, a minimum gap length of 7500 bases, and a minimum links of 3. Varying identity requirements, link ratios, and increasing linking stringencies did not change the results appreciably in this case.

The scaffolded assembly from SSPACE was polished using Pilon (https://github.com/broadinstitute/pilon/wiki). Pilon is a tool that can perform automatic improvement of draft assemblies, and can find variants among mixed samples such as strains. Pilon can detect everything from SNPs to larger indels and can also perform gap filling and detect misassembly-introducing new gaps. Further, Pilon takes advantage of pairing, orientation, and clipping information from the alignment and tracks non-valid alignments allowing for local reassessment of the context using the highest validity. For this application, Pilon was given paired Illumina 151 bps reads aligned with the BWA aligner. Since the error rate for MinION is still very high after correction, the mem algorithm was employed to allow for a large amount of the reads to be included in the alignment. This lack of stringency is not an issue as Pilon is clipping-aware, and will track and assess clipping events in a region for validity.

## Additional Information

**Accession codes:** MinION 2D read data are available for download from NCBI (BioProject: PRJNA313304).

**How to cite this article**: Deschamps, S. *et al*. Characterization, correction and *de novo* assembly of an Oxford Nanopore genomic dataset from *Agrobacterium tumefaciens.* *Sci. Rep.* **6**, 28625; doi: 10.1038/srep28625 (2016).

## Figures and Tables

**Table 1:** MinION 2D read sequencing coverage.

Sequence	no sequencing coverage	<10X sequencing coverage	Total nucleotides	% no sequencing coverage	% <10X sequencing coverage
AtPlasmid	576	8,438	556,650	0.1035	1.5159
CircularChr	2	2,421	2,772,940	0.0001	0.0873
LinearChr	5,235	13,485	2,098,034	0.2495	0.6427
TiPlasmid	994	93,310	109,974	0.9039	84.8473

Coverage is shown for all four elements of the genome: “AtPlasmid”: At plasmid; “CircularChr”: Circular chromosome; “LinearChr”: Linear chromosome; “TiPlasmid”: Ti plasmid. Number of nucleotides in the reference assembly with no sequencing coverage and <10X sequencing coverage, along with their percentage of the reference sequence of each element. Total number of nucleotides for each element is also indicated.

**Table 2:** Performance metrics of MinION 2D read correction processes.

Read Correction	Assembly	Number of Corrected Reads	Average Seq Identity %	Seq Identity % (>99%)
PBcR (Illumina Correction)	PBcR/Canu	30,152	99.9%	98.7%
Nanocorr (Illumina Correction)	SPAdes	30,406	98.5%	53.5%
PBcR (Self-Correction)	PBcR	30,173	97.3%	2.6%
Canu (Self-Correction)	Canu	28,732	99.3%	83.4%
PoreSeq Self-Correction	N/A	707	98.9%	64.8%

Read correction performed through Illumina-based correction and self-correction is shown. Corrected reads were aligned to the LBA4404 reference assembly using BWA –MEM. “Average Seq Identity %” = average sequence identity of all corrected reads to the reference assembly; “Seq Identity % (>99%)” = percentage of corrected reads exhibiting >99% sequence identity to the reference assembly in the portions of the reads aligned using BWA –MEM. “PBcR/Canu” = two unrelated assemblies were performed with PBcR and canu from the same set of Illumina-corrected reads. N/A = Not available.

**Table 3:** Summary and metrics of MinION 2D read *de novo* assemblies before and after polishing.

	Number of contigs		Contig N50 (bps)		Max Contig size (bps)		Total contig size (bps)		Seq Identity %*	
	Before polishing	After polishing	Before polishing	After polishing	Before polishing	After polishing	Before polishing	After polishing	Before polishing	After polishing
PBcR–Hybrid Assembly	22	13	616,079	673,428	2,033,791	2,384,872	5,499,815	5,499,838	>99.79%	>99.87%
PBcR–Non-hybrid Assembly	6	6	2,682,471	2,801,023	2,682,471	2,801,023	5,176,184	5,400,985	>93.71%	>98.55%
canu–Hybrid Assembly	15	13	1,378,273	1,688,510	1,690,109	1,690,462	5,490,339	5,491,108	>99.84%	>99.84%
canu–Non-hybrid Assembly	5	5	2,703,397	2,779,502	2,703,397	2,779,502	5,304,940	5,455,120	>95.45%	>97.94%
SPAdes–Hybrid Assembly	9	9	2,769,357	2,769,355	2,769,357	2,769,355	5,516,100	5,516,100	>99.96%	>99.97%

Number of contigs, contig N_50_, largest contig length (“Max contig size”) and total contig lengths (“Total contig size”) are shown. Overall sequence identity percentages (“Seq Identity %”) to the reference *Agrobacterium tumefaciens* genome assembly before and after polishing are also shown, following alignments to the reference assembly with BLASTN. Only contigs >= 1 Kbps are listed; (*) Sequence identity percentages for contigs >= 10 Kbps only.
